# Supplementary material for: Transcriptomic Insights into Mechanisms of Early Seed Maturation in the Garden Pea (Pisum sativum L.)
Source: Cells. 2020 Mar 23;9(3):779. doi: 10.3390/cells9030779 (PMC7140803; doi:10.3390/cells9030779)
Supplement: Supplementary file 1 [file cells-09-00779-s001.zip › Supplementary materials/FileS2.zip!/FileS1/Summary Sheets - Groups/Group Assignment.pdf]

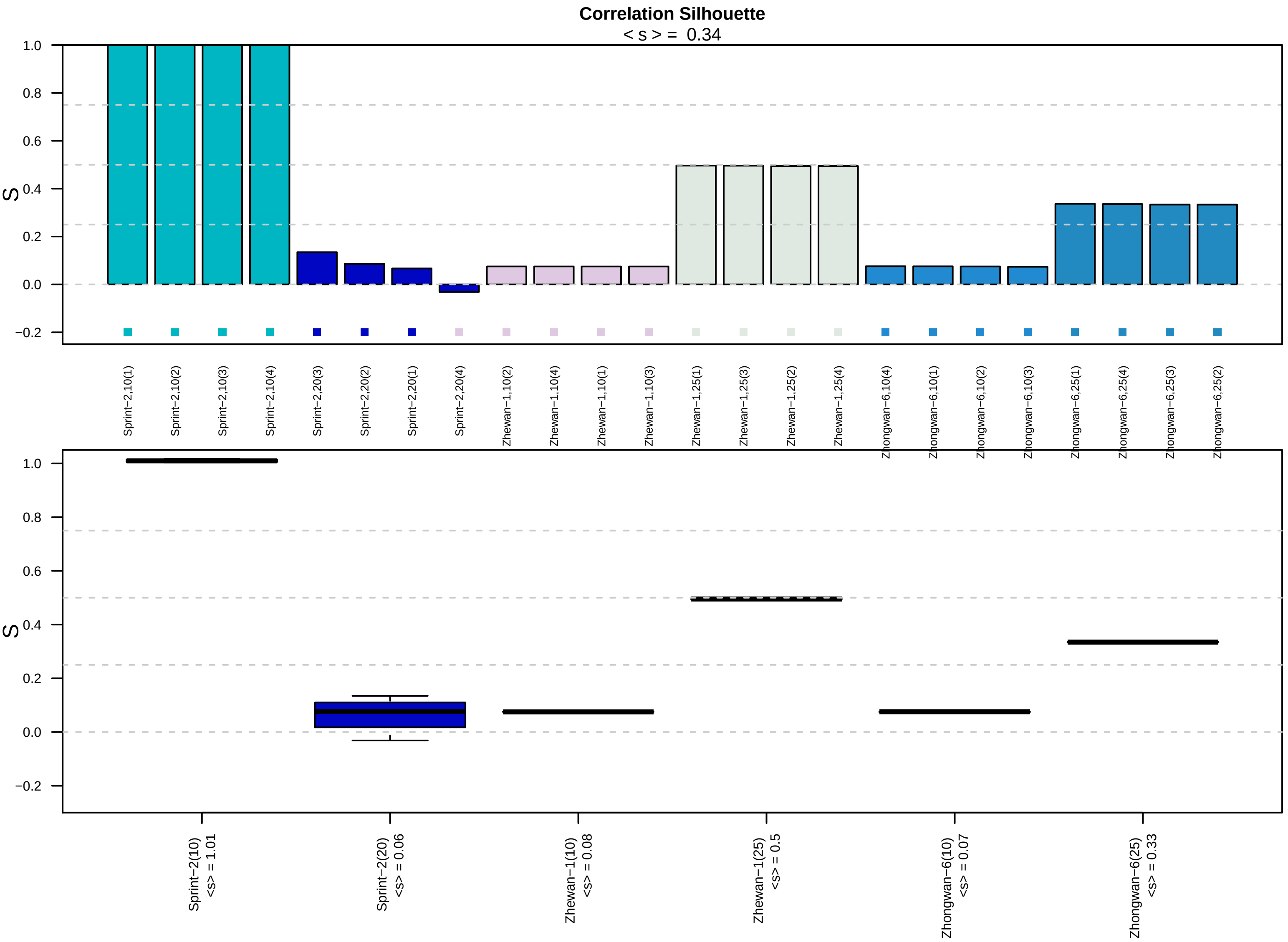

Correlation Silhouette
< s > = 0.34
s
1.0
0.8
0.6
0.4
0.2
0.0
−0.2
Sprint−2,10(1)
Sprint−2,10(2)
Sprint−2,10(3)
Sprint−2,10(4)
Sprint−2,20(3)
Sprint−2,20(2)
Sprint−2,20(1)
Sprint−2,20(4)
Zhewan−1,10(2)
Zhewan−1,10(4)
Zhewan−1,10(1)
Zhewan−1,10(3)
Zhewan−1,25(1)
Zhewan−1,25(3)
Zhewan−1,25(2)
Zhewan−1,25(4)
Zhongwan−6,10(4)
Zhongwan−6,10(1)
Zhongwan−6,10(2)
Zhongwan−6,10(3)
Zhongwan−6,25(1)
Zhongwan−6,25(4)
Zhongwan−6,25(3)
Zhongwan−6,25(2)
Sprint−2(10) <s> = 1.01
Sprint−2(20) <s> = 0.06
Zhewan−1(10) <s> = 0.08
Zhewan−1(25) <s> = 0.5
Zhongwan−6(10) <s> = 0.07
Zhongwan−6(25) <s> = 0.33

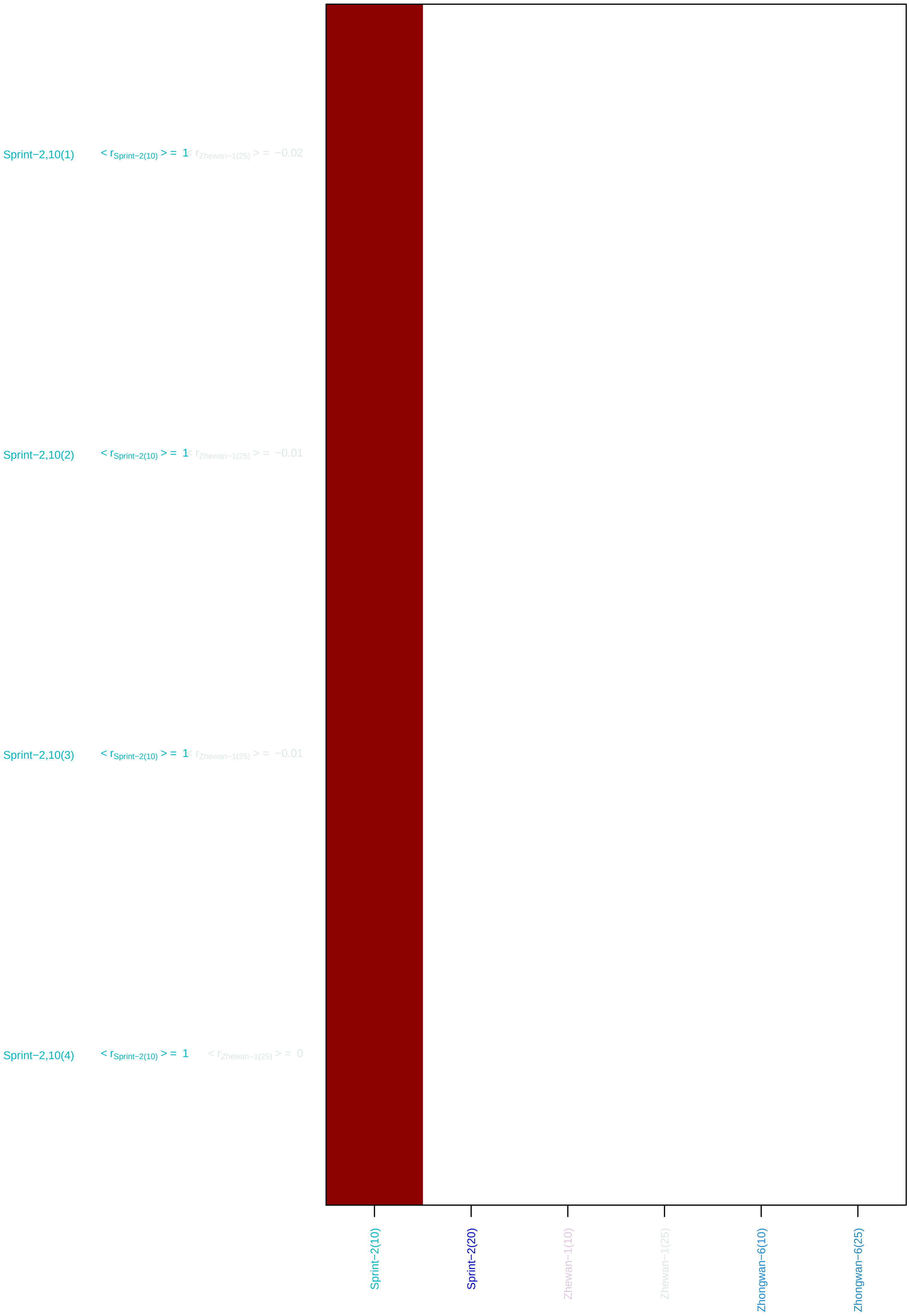
Sprint−2,10(1)
< rSprint−2(10) > = 1
< rZhewan−1(25) > = −0.02
Sprint−2,10(2)
< rSprint−2(10) > = 1
< rZhewan−1(25) > = −0.01
Sprint−2,10(3)
< rSprint−2(10) > = 1
< rZhewan−1(25) > = −0.01
Sprint−2,10(4)
< rSprint−2(10) > = 1
< rZhewan−1(25) > = 0
Sprint−2(10)
Sprint−2(20)
Zhewan−1(10)
Zhewan−1(25)
Zhongwan−6(10)
Zhongwan−6(25)

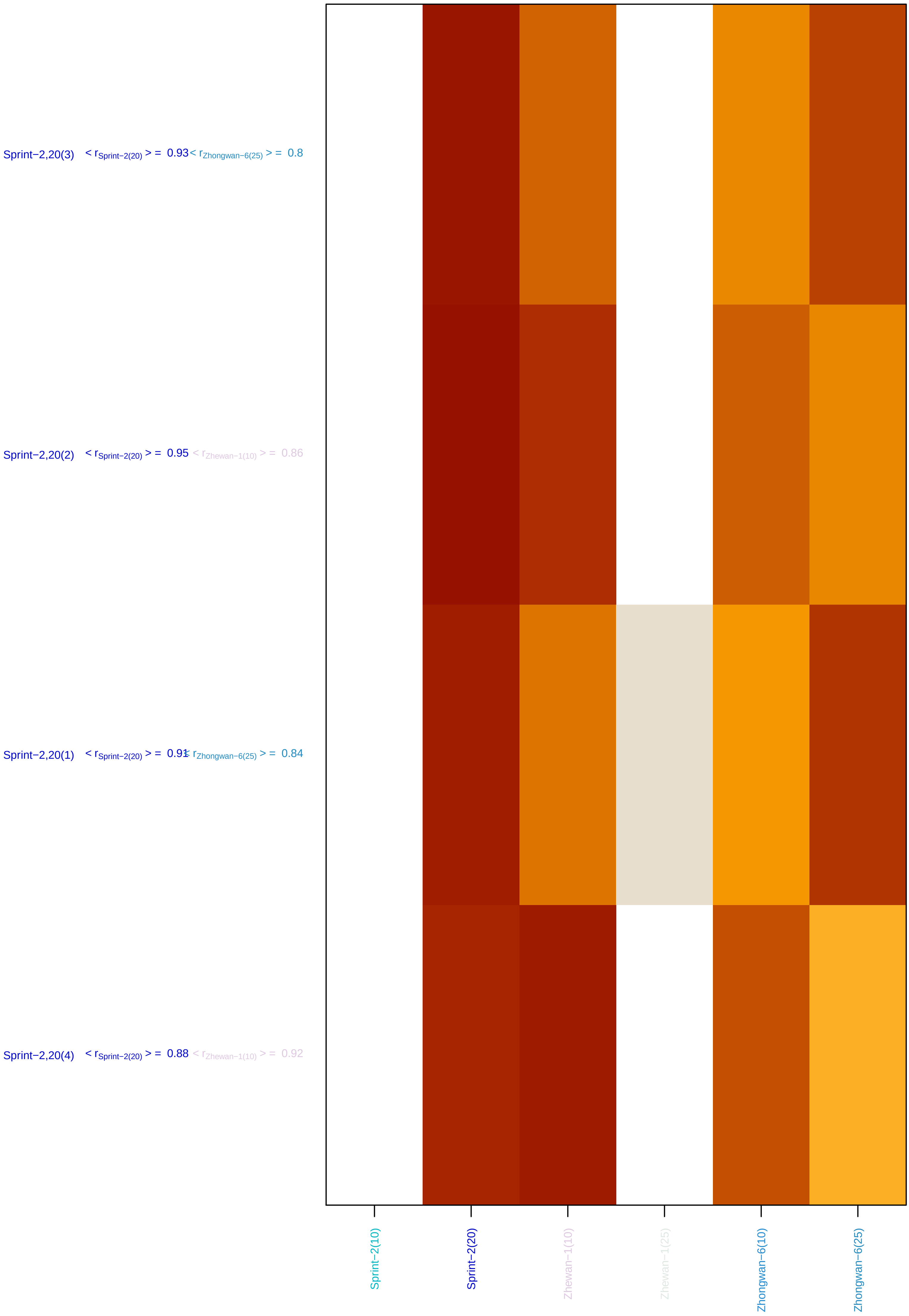
Sprint−2,20(3) < r_Sprint−2(20) > = 0.93 < r_Zhongwan−6(25) > = 0.8
Sprint−2,20(2) < r_Sprint−2(20) > = 0.95 < r_Zhewan−1(10) > = 0.86
Sprint−2,20(1) < r_Sprint−2(20) > = 0.91 < r_Zhongwan−6(25) > = 0.84
Sprint−2,20(4) < r_Sprint−2(20) > = 0.88 < r_Zhewan−1(10) > = 0.92
Sprint−2(10)
Sprint−2(20)
Zhewan−1(10)
Zhewan−1(25)
Zhongwan−6(10)
Zhongwan−6(25)

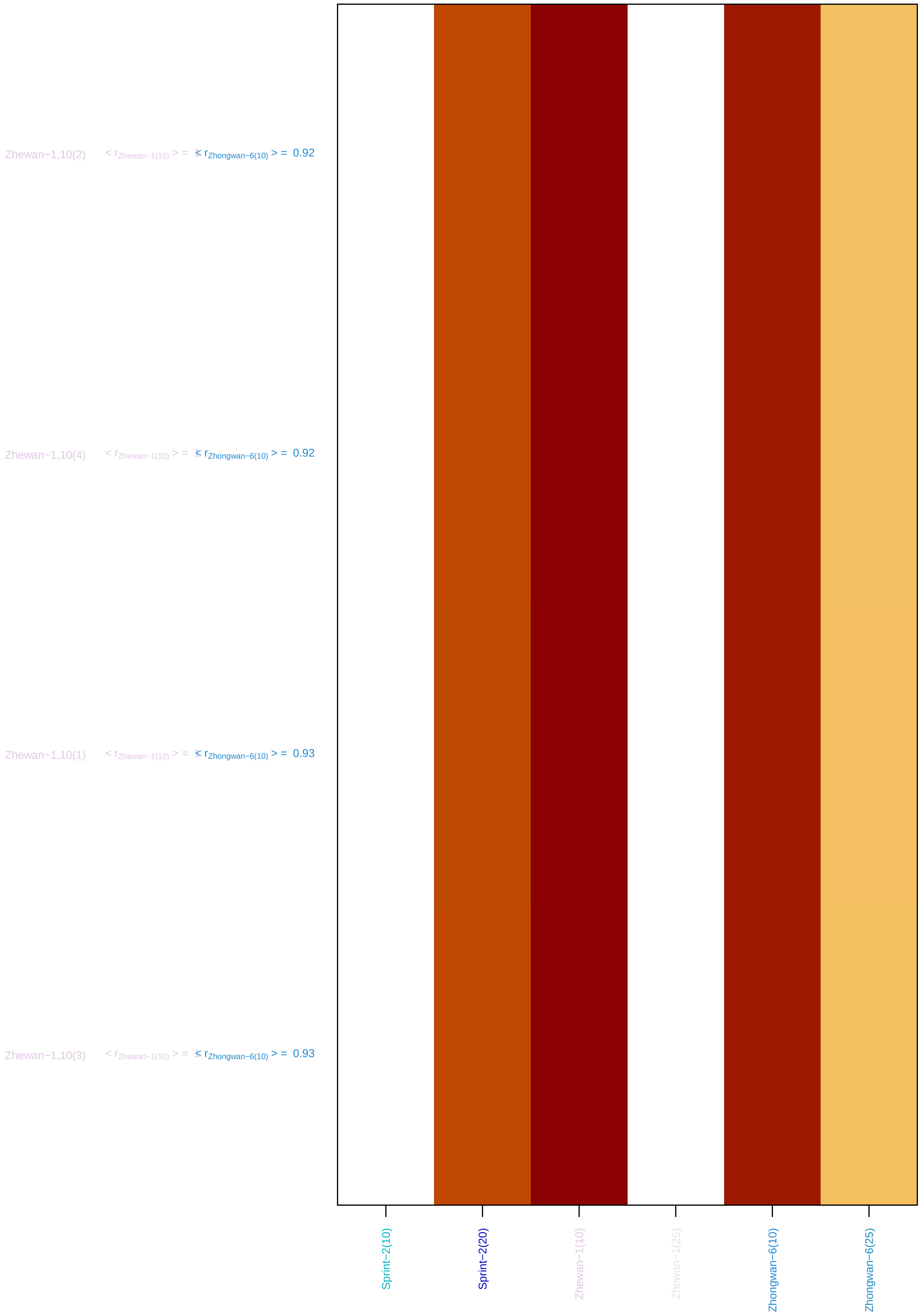
Zhewan−1,10(2)
< r Zhewan−1(10) > =
< r Zhongwan−6(10) > = 0.92
Zhewan−1,10(4)
< r Zhewan−1(10) > =
< r Zhongwan−6(10) > = 0.92
Zhewan−1,10(1)
< r Zhewan−1(10) > =
< r Zhongwan−6(10) > = 0.93
Zhewan−1,10(3)
< r Zhewan−1(10) > =
< r Zhongwan−6(10) > = 0.93
Sprint−2(10)
Sprint−2(20)
Zhewan−1(10)
Zhewan−1(25)
Zhongwan−6(10)
Zhongwan−6(25)

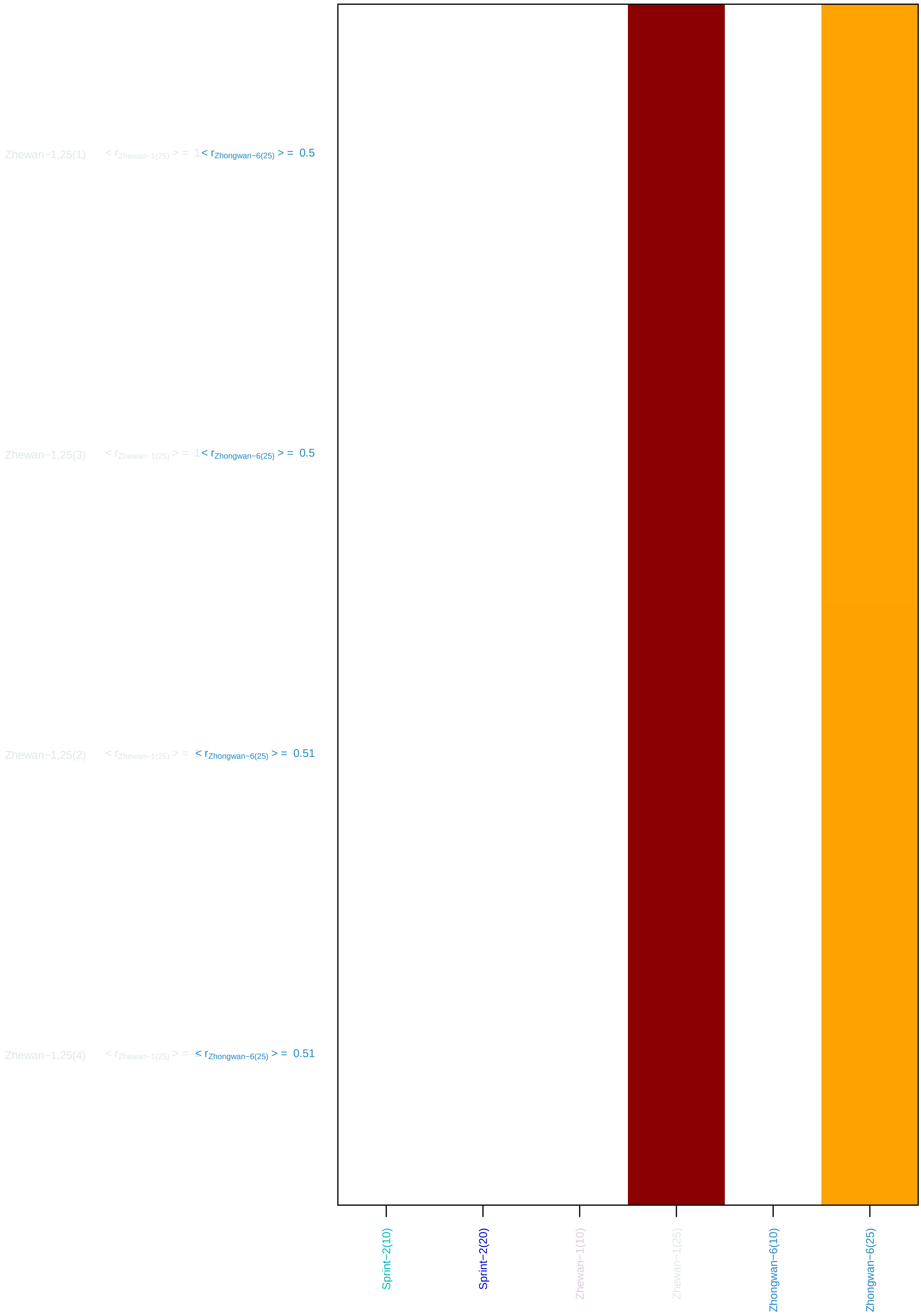
Zhewan−1,25(1)
< rZhewan−1(25) > = 1 < rZhongwan−6(25) > = 0.5
Zhewan−1,25(3)
< rZhewan−1(25) > = 1 < rZhongwan−6(25) > = 0.5
Zhewan−1,25(2)
< rZhewan−1(25) > = < rZhongwan−6(25) > = 0.51
Zhewan−1,25(4)
< rZhewan−1(25) > = < rZhongwan−6(25) > = 0.51
Sprint−2(10)
Sprint−2(20)
Zhewan−1(10)
Zhewan−1(25)
Zhongwan−6(10)
Zhongwan−6(25)

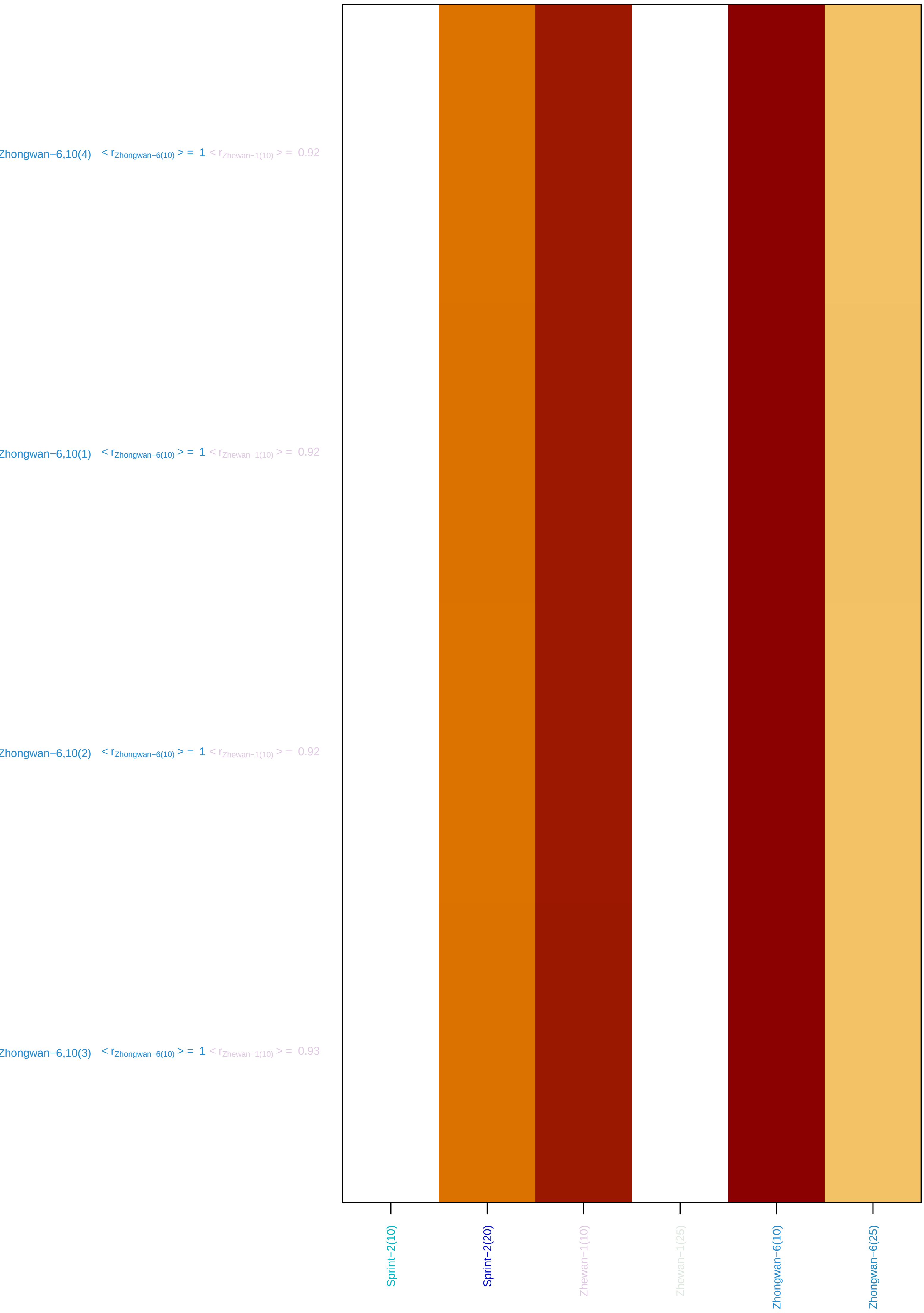
Zhongwan−6,10(4) < r Zhongwan−6(10) > = 1 < r Zhewan−1(10) > = 0.92
Zhongwan−6,10(1) < r Zhongwan−6(10) > = 1 < r Zhewan−1(10) > = 0.92
Zhongwan−6,10(2) < r Zhongwan−6(10) > = 1 < r Zhewan−1(10) > = 0.92
Zhongwan−6,10(3) < r Zhongwan−6(10) > = 1 < r Zhewan−1(10) > = 0.93
Sprint−2(10)
Sprint−2(20)
Zhewan−1(10)
Zhewan−1(25)
Zhongwan−6(10)
Zhongwan−6(25)

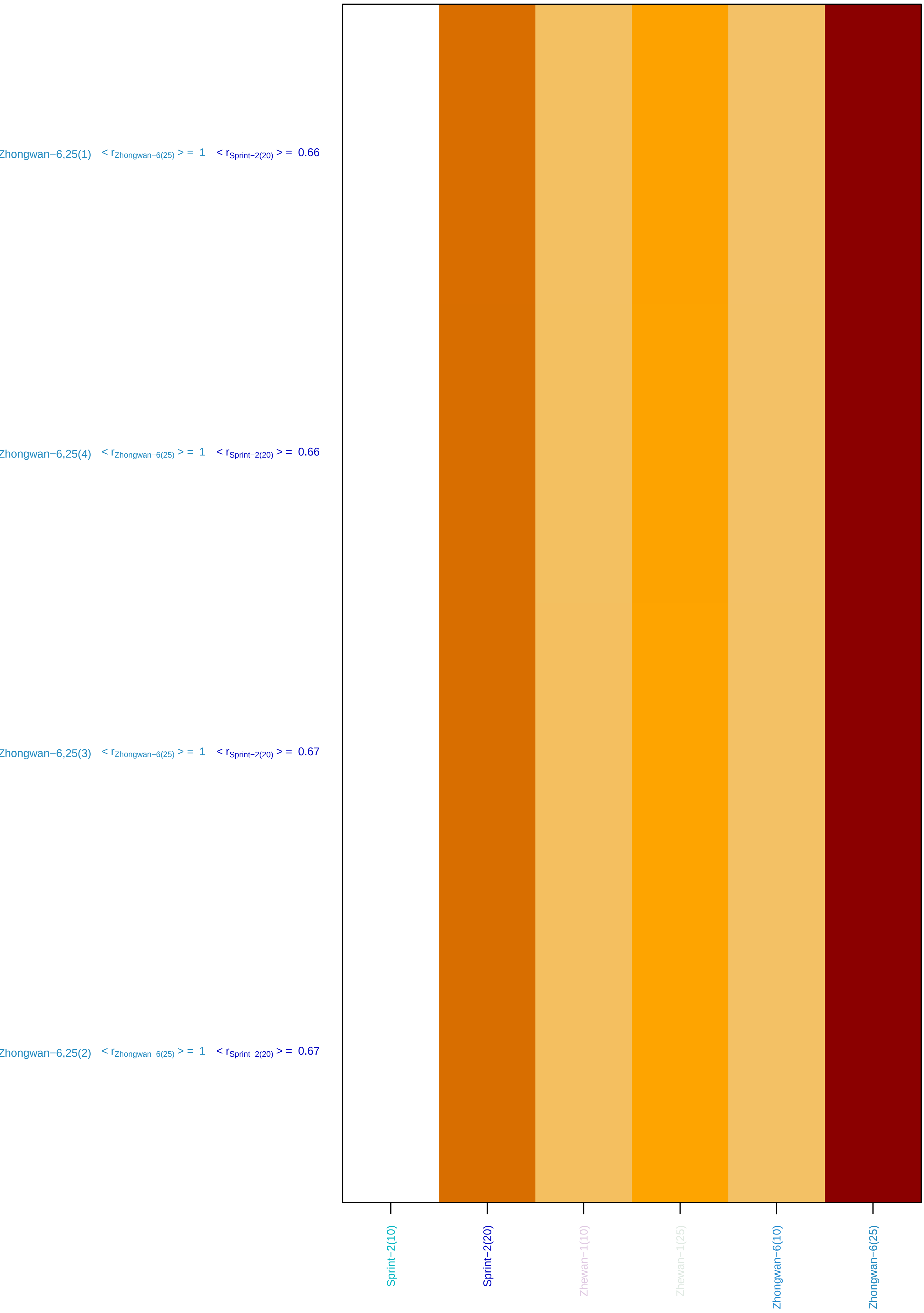
Zhongwan−6,25(1) < r_Zhongwan−6(25) > = 1 < r_Sprint−2(20) > = 0.66
Zhongwan−6,25(4) < r_Zhongwan−6(25) > = 1 < r_Sprint−2(20) > = 0.66
Zhongwan−6,25(3) < r_Zhongwan−6(25) > = 1 < r_Sprint−2(20) > = 0.67
Zhongwan−6,25(2) < r_Zhongwan−6(25) > = 1 < r_Sprint−2(20) > = 0.67
Sprint−2(10)
Sprint−2(20)
Zhewan−1(10)
Zhewan−1(25)
Zhongwan−6(10)
Zhongwan−6(25)
